# Mechanistic insight of the potential of geraniol against Alzheimer’s disease

**DOI:** 10.1186/s40001-022-00699-8

**Published:** 2022-06-14

**Authors:** Ying Liu, Shujing Zhou, Xufeng Huang, Hafiz Muzzammel Rehman

**Affiliations:** 1 Department of Cardiology, 6th Medical Centre, Chinese PLA General Hospital, Beijing, China; 2 Faculty of Medicine, University of Debrecen, Debrecen, 4032 Hungary; 3 Faculty of Dentistry, University of Debrecen, Debrecen, 4032 Hungary; 4 School of Biochemistry and Biotechnology, University of the Punjab, Lahore, 54590 Punjab Pakistan; 5 Alnoorians Group of Institutes, 55-Elahi Bukhsh Park, Amir Road, Shad Bagh, Lahore, 54000 Pakistan

**Keywords:** Network modeling, Drug discovery, Data integration, Geraniol, Alzheimer’s disease

## Abstract

**Background:**

Alzheimer’s disease (AD) as a neurodegenerative disease occupies 3/5–4/5 cases among patients with dementia, yet its pathogenetic mechanism remains unclear. Geraniol, on the other hand, is a well-known extract from essential oils of aromatic plants and has been proven that it has outstanding neuroprotective effects as well as ameliorating influence in memory impairment. Therefore, the present study aims to elucidate the potential of geraniol against AD by network pharmacology-based approach combined with molecular modeling study.

**Materials and methods:**

Firstly, we evaluated the druggability of geraniol by ADME method. Then, we obtained the geraniol targets and AD-related targets from multiple open data sources. Afterward, we calculated the intersection through a Venn diagram to find common targets, and via Panther classification system to categorize them. In order to gain a macroscopic understanding of these common targets, we carried out GO terms and KEGG pathways enrichment analyses, according to which we constructed a compound–target–pathway–disease network. In addition, we built a preliminary PPI network which was further analyzed both functionally and topologically. Consequently, five hub targets were sorted out. Finally, we conducted molecular docking and molecular dynamic simulation to validate our findings.

**Results:**

In the present study, the pharmacological properties of geraniol were assessed according to ADME and Lipinski’s rule, which demonstrate promising druggability. Then, from 10,972 AD-related targets and 33 geraniol targets, 29 common targets were identified, among which 38.1% of them are metabolite interconversion enzymes, 23.8% are protein modifying enzymes, 33.3% are transmembrane receptors, and the rest are transporters. Enrichment analyses hint that geraniol is involved in cholinergic synapse, serotonergic synapse, and neuroactive ligand–receptor interaction. We also built a preliminary PPI network to investigate the interplay between these targets and their extensive interactions. Then, by functionally clustering the preliminary PPI network, we gained a cluster of proteins which formed a subnetwork with score of 8.476, and 22 nodes. Its results of GO terms and KEGG pathways enrichment analyses once again suggests that geraniol actively participates in cholinergic synapse, serotonergic synapse, and neuroactive ligand–receptor interaction, which are believed to be strongly associated with AD pathogenesis. Besides, topological analyses of the preliminary PPI network helped find 5 hub targets (i.e., CHRM3, PRKCA, PRKCD, JAK1, JAK2). To verify their interaction with geraniol molecule, we conducted molecular docking, and found that CHRM3 possesses the highest affinity in binding, indicating that geraniol molecules are closely bound to each hub target, and CHRM3 may serve as a key target of geraniol against AD. It was then further confirmed by molecular dynamic simulation, the result of which supports our hypothesis.

**Conclusion:**

The present study shares a mechanistic insight of the potential of geraniol against AD, giving a reference to future experimental studies.

**Supplementary Information:**

The online version contains supplementary material available at 10.1186/s40001-022-00699-8.

## Introduction

Alzheimer’s disease (AD) has been a common neurodegenerative disease that occupies 3/5–4/5 cases among patients with dementia, yet its pathogenetic mechanism of AD is very complicated and thus remains unclear [1, 2]. The current hypotheses include Aβ amyloid cascade theory, the theory of microtubule-associated protein abnormalities, the theory of central cholinergic damage, the theory of infection, the abnormal growth of gut microbes, etc. [3–7]. Due to the blood–brain barrier, most of the drugs such as steroid hormones have limited therapeutic effects on AD, which urges researchers to develop new drugs for its prevention and treatment [8]. On the other hand, thanks to the rapid development of modern bioinformatics, publicly accessible databases provide an unprecedented wealth of information to help drug discovery. By combining data available in these databases with the proper bioinformatical tools, we can elucidate the molecular targets of natural compounds [9]. One such molecule is geraniol, a well-known component of essential oils of aromatic plants and has been proven possessing outstanding neuroprotective effects as well as ameliorating influence in memory impairment [10–13]. Therefore, the present study aims to elucidate the potential of geraniol against AD by network pharmacology-based approach combined with molecular modeling study.

**Fig. 1:**
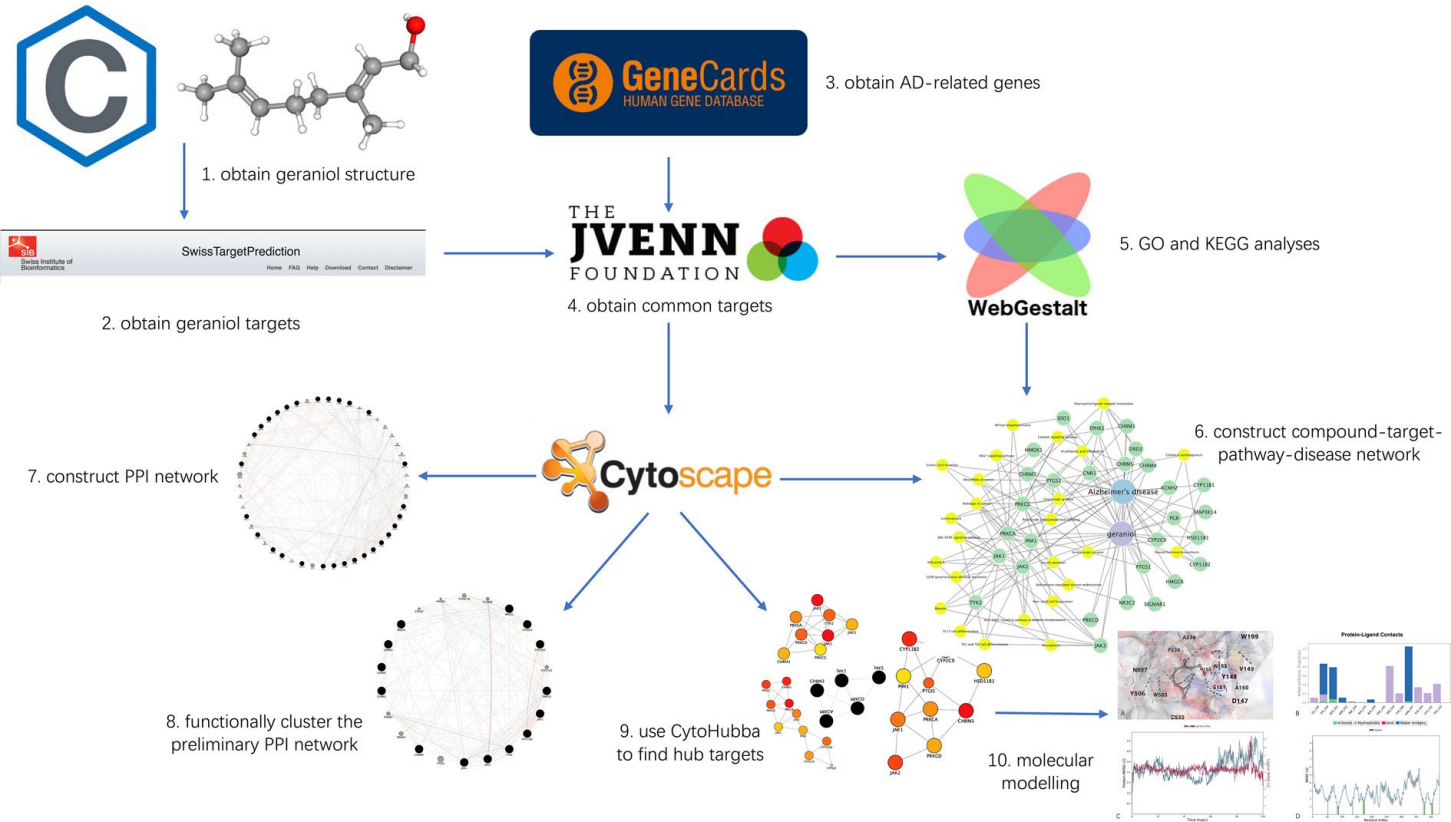
Workflow of the present study

Figure 1 demonstrates the workflow of the present study in a graphical manner.

## Materials and methods

### Druggability assessment

The sdf file of geraniol’s structure was downloaded from PubChem (https://pubchem.ncbi.nlm.nih.gov/) and was input into SwissADME (www.swissadme.ch) [14] that provides literature and structure-based pharmacological information of geraniol, including molecular weight, number of H-bond acceptors and donors, topological polar surface area, GI absorption, blood–brain barrier, Lipinski’s rule, etc. From this server, we obtained the corresponding pharmacological properties for ADME and Lipinski’s rule evaluation.

### Target identification

We used the sdf file again, but with Swiss Target Predictor (www.swisstargetprediction.ch) this time to find the putative targets of geraniol [15]. Then, we searched from GeneCard (https://www.genecards.org) to obtain the AD-related targets [16]. Afterward, we used the JVenn program (http://jvenn.toulouse.inra.fr/app/example.html) to calculate the intersection of them which are their common targets [17]. These targets were then classified by the Panther classification system (http://www.pantherdb.org/) [18].

### Enrichment analyses of the common targets

WebGestalt (www.webgestalt.org), as a robust online bioinformatic toolkit, was used for GO and KEGG pathway enrichment analyses of the common targets [19].

### Network construction

Based on the results, we constructed the geraniol–target–pathway–AD network by Cytoscape (v3.9.0) for better visualization [20]. In addition, we exerted the list of common targets into GeneMANIA, a Cytoscape plug-in, to build a preliminary protein–protein interaction (PPI) network [21].

#### Functional clustering and in-depth enrichment analyses of the preliminary PPI network

We used a Cytoscape plug-in, “MCODE” to functionally cluster the preliminary PPI network, which created a subnetwork [22]. With the newly created subnetwork, we conducted GO and KEGG analyses again to gain a more comprehensive understanding of the role that geraniol may play in the mechanism against AD.

#### Hub target screening via topological analysis of the preliminary PPI network

We used another Cytoscape plug-in, “CytoHubba”, to extract a core PPI network that frames the preliminary PPI network via intersectional merge of the subnetworks created from degree, closeness, and betweenness methods [23].

### Molecular docking verification

To predict the interaction between the targets and the compounds, we downloaded the crystal structures of the 5 hub targets from PDB library (i.e., 3egy, daj, 2b7a, 3iw4, 1yrk) and then docked them with geraniol molecule on CB-dock platform (http://clab.labshare.cn/cb-dock/php/blinddock.php) based on AutoDock Vina [24, 25]. Afterward, we obtained the corresponding affinity energy values and the binding sites, the center, and the customized docking box size. To further validate the binding mode, we performed molecular dynamic simulation. The simulation was performed by Desmond at 100 ns to investigate the binding conformational stability of the protein–ligand complex [26]. The stability of the protein–ligand complex was observed to be maintained during the whole 100 ns simulation for compounds based on RMSD, RMSF, and hydrogen bond interactions.

## Results

### Druggability assessment

As usually a molecule fulfilling the parameters of RBN < 10, OB ≥ 20%, DL ≥ 0.1, TPSA < 60 Å^2^, and Lipinski’s rule of five (i.e., MW < 500 Da, AlogP < 5, Hdon < 5, Hacc < 10) is considered as potential druggable substance, geraniol possessing MW = 154.25 Da, Hdom = 1, Hacc = 1, RBN = 4, TPSA = 20.23 Å^2^, LogKp = − 4.71 cm/s, therefore is deemed to be one of such molecules that can be further optimized into orally administered drug.

Details are shown in Table 1.

**Table 1:** Basic pharmacological data of geraniol

Name	MW (Da)	Hdom	Hacc	RBN	TPSA (Å^2^)	GI	BBB	LogKp (cm/s)	Lipinski
Geraniol	154.25	1	1	4	20.23	High	Yes	− 4.71	No violation

*MW* molecular weight, *Hdon* hydrogen donor, *Hacc* hydrogen acceptor, *RBN* number of bonds that can perform free rotation, *TPSA* surface sum over all polar atoms, primarily oxygen and nitrogen, also including their attached hydrogens, *GI absorp* gastrointestinal absorption, *BBB* blood–brain barrier, *LogKp* an improved measurement of skin permeation coefficient, *Lipinski* Lipinski’s rule of five, an empirical rule to evaluate whether a molecule is suitable to be developed into orally administrated drug

### Target identification

**Fig. 2:**
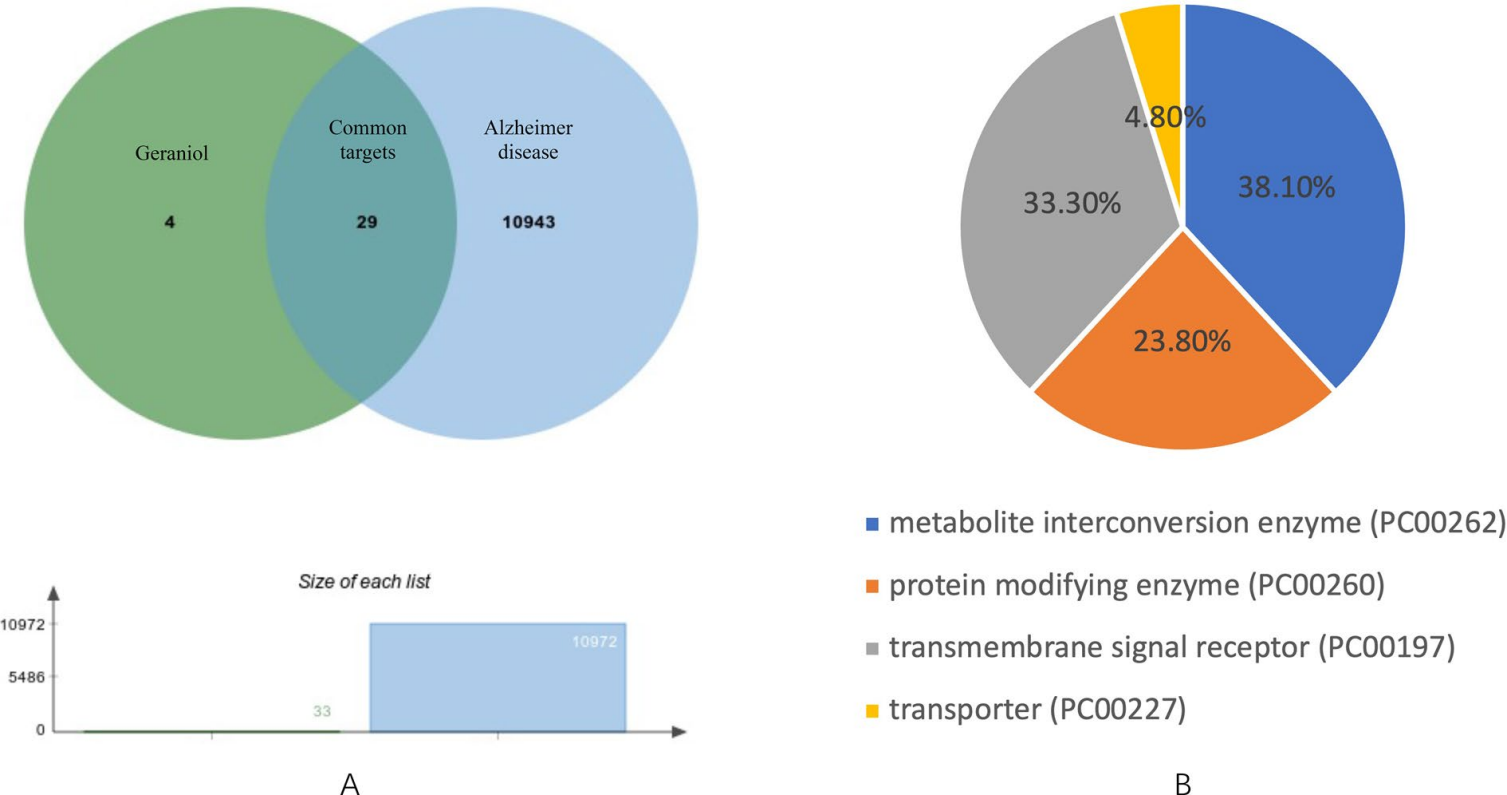
Identification of targets. **A** Venn diagram showing common targets between geraniol and AD. The green circle represents geraniol targets, the blue circle represents AD targets. **B** Pie chart showing different categories of the common targets with corresponding colors and percentages

Through searching, 10,972 AD-related targets and 33 geraniol targets were found from which 29 of them are common targets. They are demonstrated as an intersection in the Venn diagram in Fig. 2A. After being categorized by the Panther classification system, it appears that 38.1% of them are metabolite interconversion enzymes, 23.8% are protein modifying enzymes, 33.3% are transmembrane receptors, and the rest are transporters. The percentage of each category is indicated in the pie chart in Fig. 2B.

### Enrichment analyses of the common targets

**Fig. 3:**
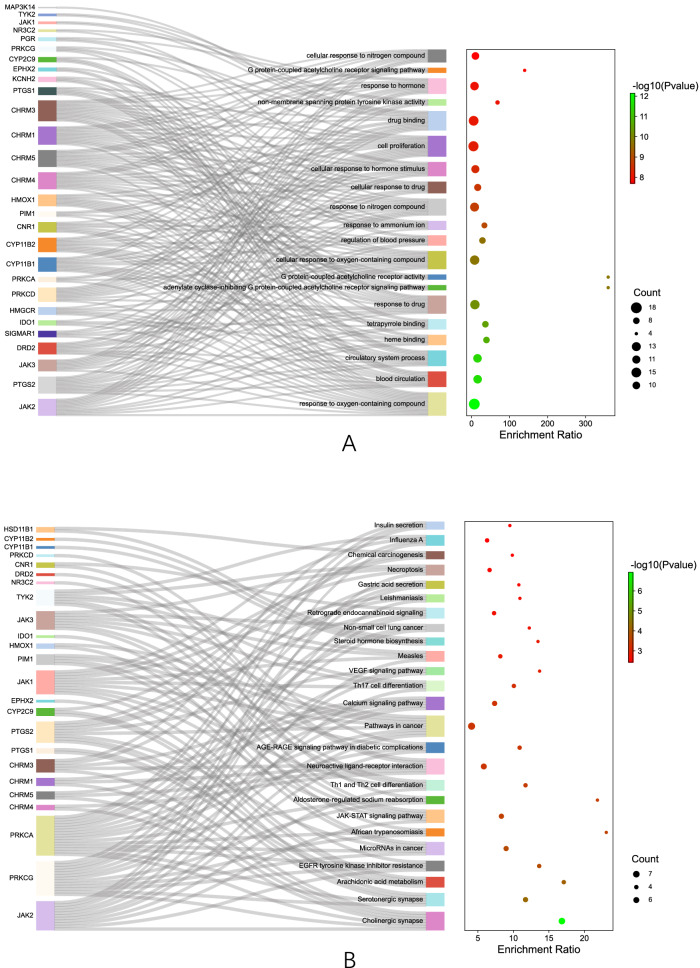
Enrichment analyses of the common targets. **A** Bubble plot combined with Sankey diagram demonstrating the top 20 most enriched GO terms. **B** Bubble plot combined with Sankey diagram demonstrating the top 20 most enriched KEGG pathways

With the common targets, we conducted GO terms and KEGG pathways enrichment analyses to gain a macroscopic understanding of their functions, from which we found that GO:1901700 (response to oxygen-containing compound) is the most enriched GO term, followed by GO:0008015 (blood circulation), GO:0003013 (circulatory system process), and hsa04725 (cholinergic synapse), hsa04726 (serotonergic synapse), hsa00590 (arachidonic acid metabolism) are the most enriched KEGG pathways, as shown in Fig. 3A, B. The rare data of the enrichment analyses can be visited in the Additional file 1 S2_Rare data for GO terms and KEGG pathways analyses.

### Network construction

**Fig. 4:**
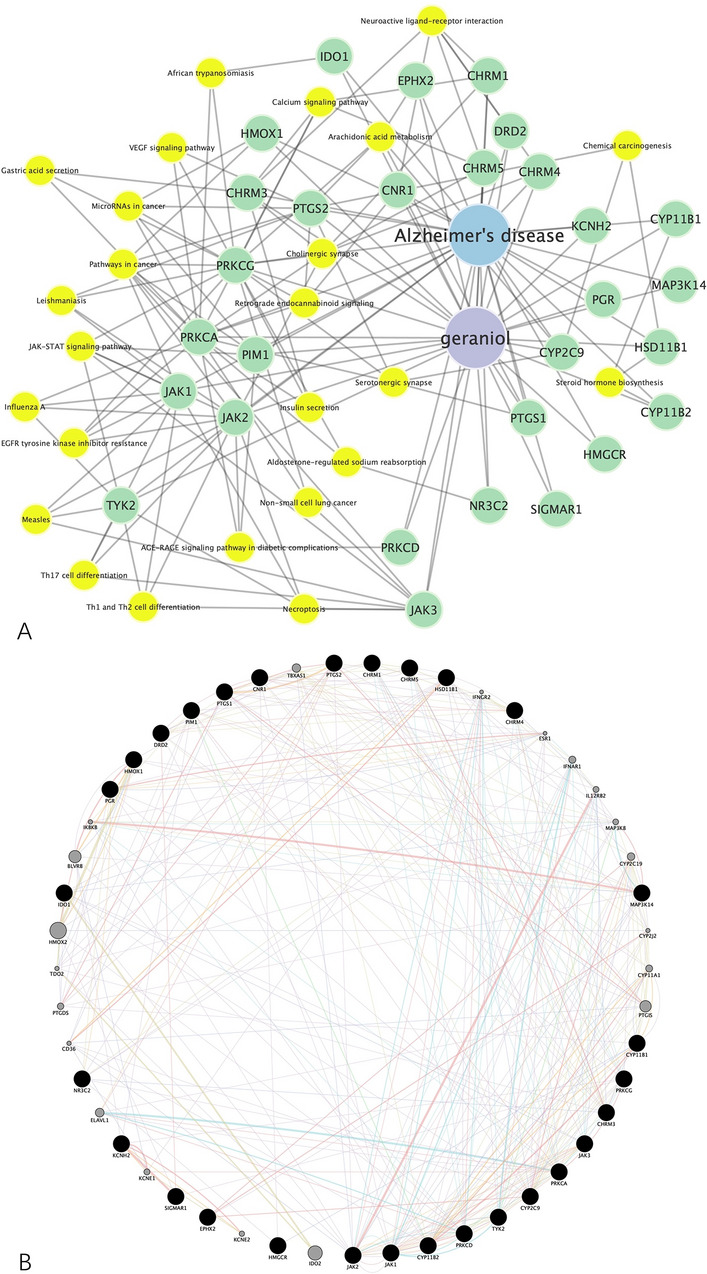
Construction of network. **A** Geraniol–target–pathway–AD network. The purple node represents geraniol, the blue node represents AD, the green nodes represent their common targets, the yellow nodes represent their related KEGG pathways. **B** Preliminary protein–protein interaction (PPI) network organized in degree sorted circular layout. The higher the degree of the node in the network, the larger the node and the darker the color

Based on the enrichment results, we constructed the geraniol–target–pathway–AD network, as shown in Fig. 4A, with 56 nodes and 165 edges. For the data used during the construction of the network, please visit the Additional file 1 S1_Rare data for target identification. The characteristic path length is 2.421, network density is 0.107, the heterogeneity is 0.938, the network centralization is 0.436. In addition, in Fig. 4B, we built a PPI network for deeper analyses to see the logic behind. The preliminary PPI network contains 49 nodes and 317 edges, with 2.087 characteristic path length, 0.190 network density, 0.367 network heterogeneity, and 0.106 network centralization.

#### Functional clustering and in-depth enrichment analyses of the preliminary PPI network

**Fig. 5:**
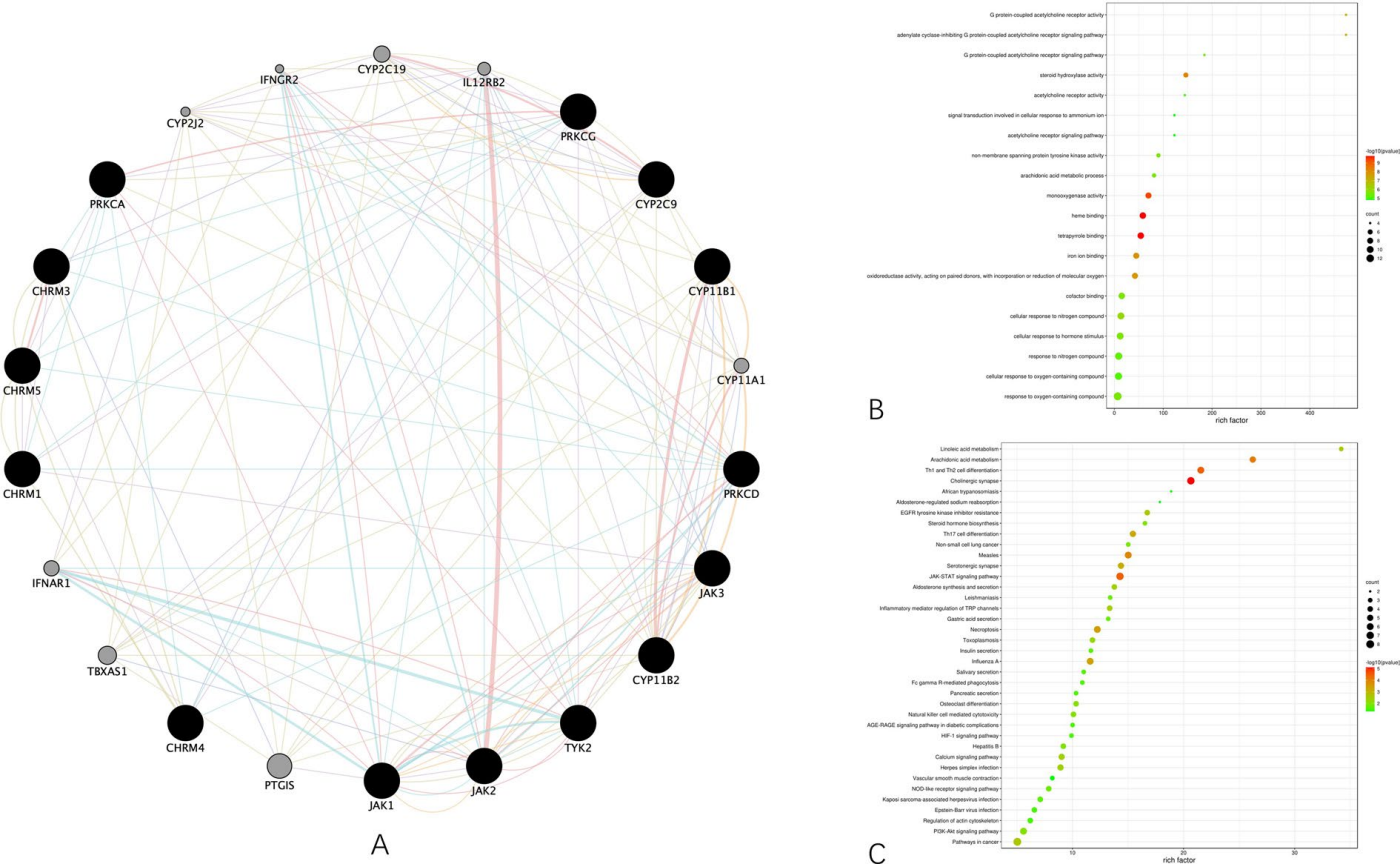
Functional clustering and corresponding GO terms and KEGG pathways enrichment analyses. **A** The functional cluster detected from the preliminary PPI network by MCODE. **B** Bubble plot showing top 20 most enriched KEGG pathways. FDR values are represented by colors, gene counts are represented by bubble size. **C** Bubble plot showing the top 20 most enriched GO terms. FDR values are represented by colors, gene counts are represented by bubble size

By setting *K* core value to 3 and keeping the rest parameters in default values, we functionally clustered the proteins involved in the preliminary PPI network as what Fig. 5A shows. The modulation score of the cluster is 8.476 which consists of 22 nodes representing 22 genes.

Based on these genes, we conducted the GO terms and KEGG pathways analyses to have a deeper insight of their functions. The results in Fig. 5B, C further suggest that geraniol may be against AD by participating in cholinergic synapse, serotonergic synapse, and neuroactive ligand–receptor interaction, as these pathways have more genes involved. The rare data of the enrichment analyses can be visited in the Additional file 1 S3_Rare data for GO terms and KEGG pathway analyses of functional clustering.

#### Hub target screening via topological analysis of the preliminary PPI network

**Fig. 6:**
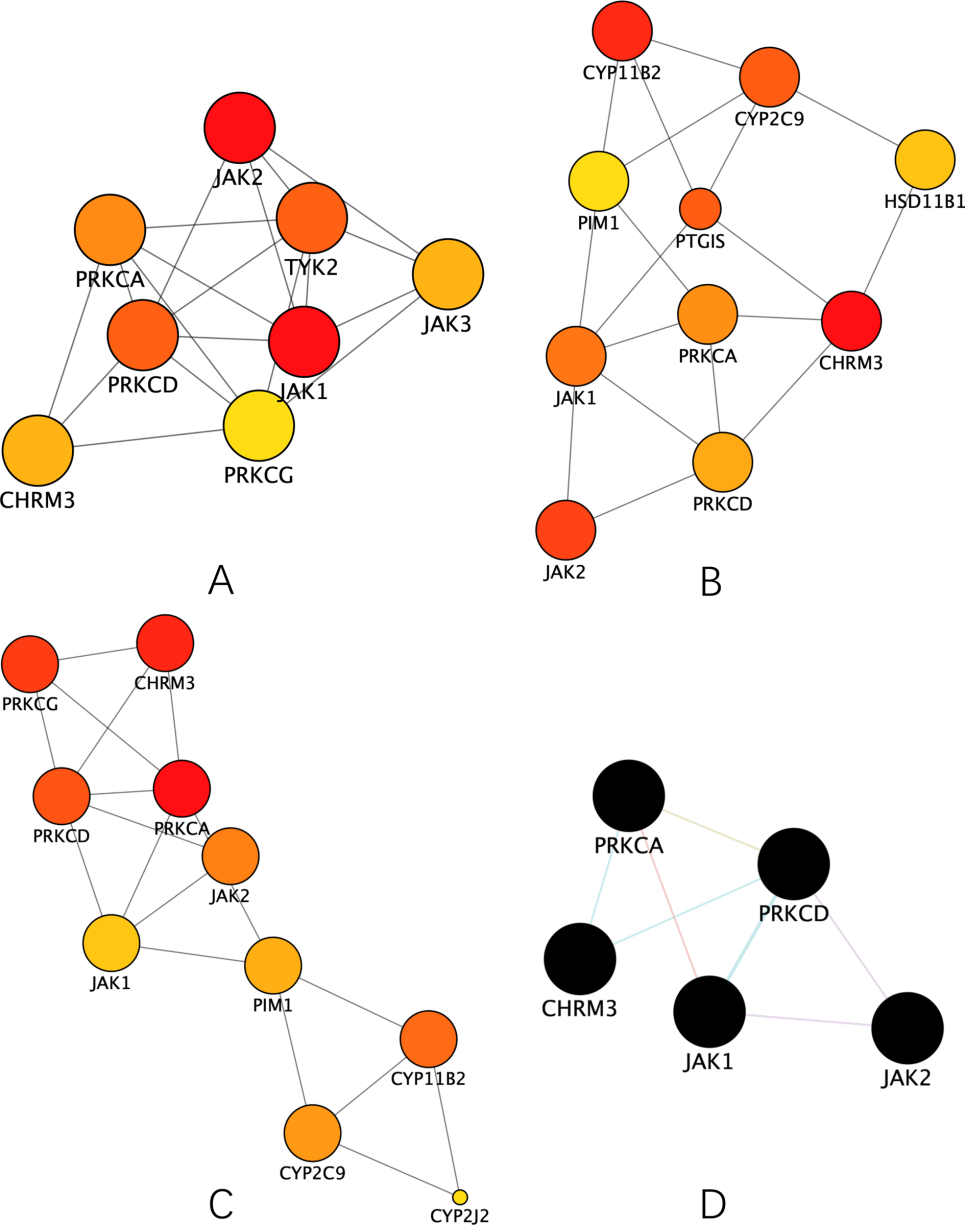
Screening of the hub targets from preliminary PPI network. **A** Subnetwork extracted from the preliminary PPI network based on degree method. Top 10 targets were selected, among which 2 targets were found to have no connection with the others (thus they were removed). **B** Subnetwork extracted from the preliminary PPI network based on closeness method. **C** Subnetwork extracted from the preliminary PPI network based on betweenness method. **D** Core subnetwork merged from the intersection of the previous subnetworks

By using the CytoHubba plug-in, we can make a further in-depth analysis of the topology of the network. We screen 3 subnetworks with top 10 ranking nodes in Fig. 6A–C based on degree, closeness, betweenness methods, respectively. Then, these subnetworks underwent intersectional merge, creating a core PPI subnetwork in Fig. 6D composed of 5 hub targets: JAK1, JAK2, PRKCD, PRKCA, CHRM3.

### Molecular docking and molecular dynamic simulation

**Fig. 7:**
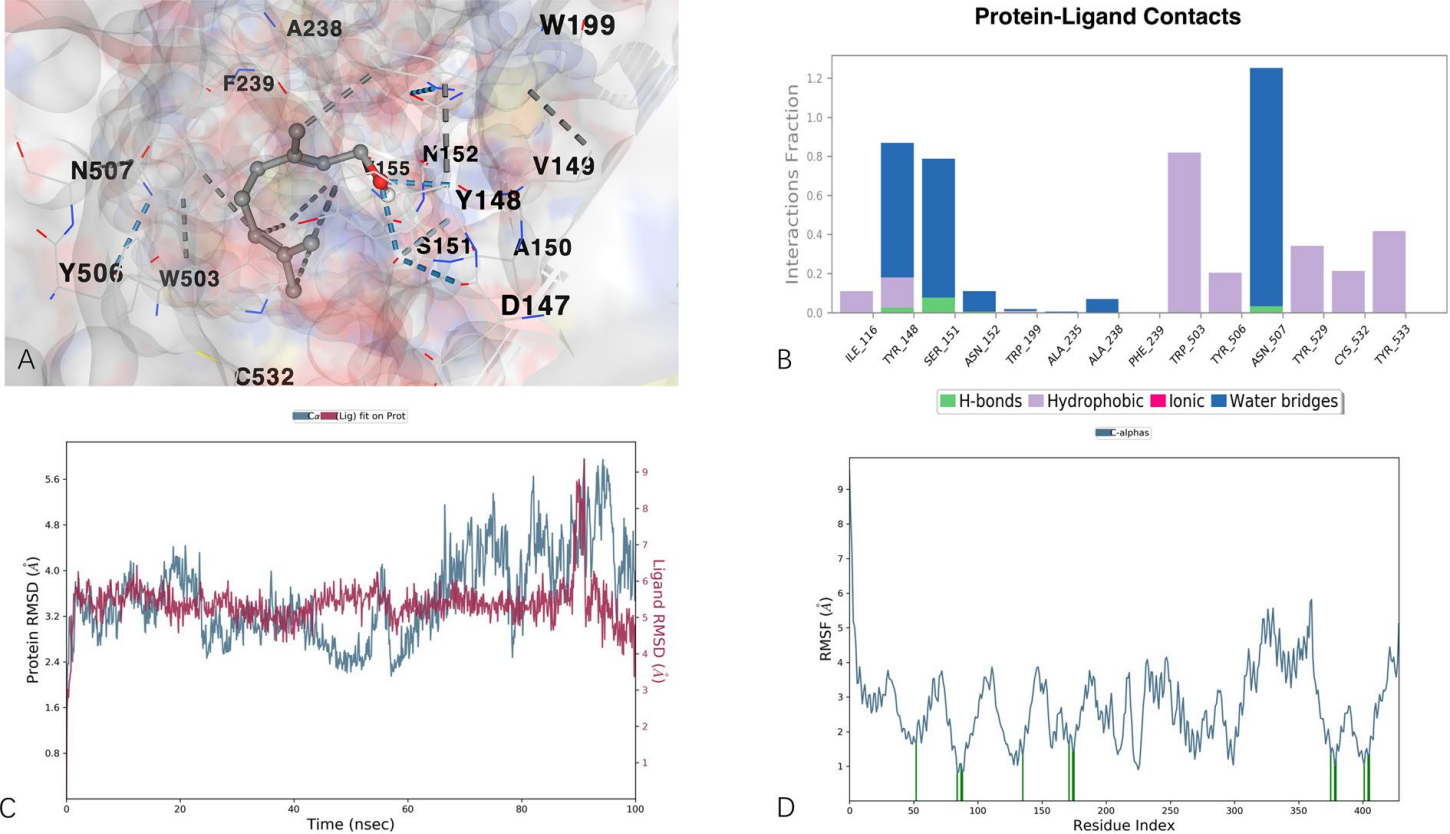
Molecular docking and molecular dynamic simulation. **A** Three-dimensional demonstration of geraniol–CHRM3 complex. **B** Selected important protein–ligand contact points. **C** RMSD diagram of in 100 ns during molecular dynamic simulation. **D** RMSF diagram during molecular dynamic simulation

Molecular docking between geraniol and the hub targets was carried out to ascertain the binding mode between them. It was found that the best run came with CHRM3 with the lowest affinity energy of − 5.9 kcal/mol, followed by JAK1, JAK2 and PRKCA (all of them are − 5.8 kcal/mol), and PRKCD (− 4.8 kcal/mol), which suggests that CHRM3 may serve as the most important player of the underlying mechanism against AD. Detailed of the binding modes are available in the Additional file 1 S4_Rare data for molecular docking. We further verified the binding mode between CHRM3 and geraniol through molecular dynamic simulation which was performed on the top hit containing high binding energies. Over the simulation period, the projected conformational changes from the initial structure were presented in terms of root mean square deviation (RMSD). Moreover, structural stability, atomic mobility, and residue flexibility at times of interaction of protein-hit were expressed with root mean square fluctuation (RMSF) values. The RMSD values for protein–ligand complex were calculated and given in Fig. 7B. RMSD of the complex showed deviation of about 2 Å at 90 ns and then there was no significant fluctuation and the simulation converges, suggesting a good stability of the protein–ligand complex. For RMSF, there was not much fluctuation observed and the structure was stabilized comparatively and there was no fluctuation where ligand made contacts with protein, as shown in Fig. 7C. Overall, the ligand showed significant different types of intermolecular interactions during the entire simulation including hydrogen bonds, ionic, water bridges, and hydrophobic. The residues participating in these interactions include ILE 116, TYR 148, SER 151, ASN 152, TRP 199, ALA 235, PHE 239, TRP 503, TYR 506, ASN 507, TYR 529, ASN 507, TYR 529, CYS 532 and TYR 533, as shown in Fig. 7D.

## Discussion

Although there has been numerous research on geraniol and AD separately, to the best of our knowledge, the present study is the very first one to integrate network pharmacology and molecular modeling to give a mechanistic insight of the potential mechanisms of geraniol in AD treatment.

The network pharmacological analysis identified 10,972 AD-related targets, 33 geraniol targets, among which 29 of them are common targets. Among the 29 common targets, 38.1% of them are metabolite interconversion enzymes, 23.8% are protein modifying enzymes, 33.3% are transmembrane receptors, and the rest are transporters. They mainly participate in cholinergic synapse, serotonergic synapse, and neuroactive ligand–receptor interaction. Earlier progress in neuroscience revealed that high density of cholinergic synapse in the human central nervous system is critically important in terms of memory, learning, as well as attention and thus significantly associated with age-dependent neurological declines, such as AD [6]. Dysregulation of serotonergic synapse was argued to be a major cause of the AD in recent years (i.e., the serotonergic hypothesis) [27]. Therefore, it is thought that geraniol may exert its effects against AD through such neurology-related pathways.

In addition, we constructed a preliminary PPI network aiming to sort out the hub targets. After topological optimization of the network, 5 hub targets (i.e., CHRM3, PRKCA, PRKCD, JAK, JAK2) were screened out. CHRM3 has been found to heterodimerize with CHRM2 which contributes to memory and cognition [11–13, 28]. PRKCA is said to be related to amyloid protein formation in AD [29–31]. Besides, previous scientific research has reported the dual role of PRKCD in proapoptotic kinase activation and apoptotic caspase cascade activation, which is important in the pathogenesis of neurodegenerative disorders including AD [32]. Clinical studies have also shown that dysregulation of the expression of JAK1 and JAK2 associates with brain inflammatory processes and neuronal or glial survival, which are consequently involved in most brain disorders including the pathogenesis of AD [33]. In short, the 5 hub targets are in strong correlation with AD pathogenesis and deterioration.

Furthermore, we conducted molecular modeling to study the robustness of the interaction between each hub target and geraniol molecule. Subsequently, it is found that CHRM3 possesses the highest affinity with geraniol molecule and thus may serve as the key target of geraniol molecule in AD treatment.

In conclusion, the present study shares a mechanistic insight of the potential of geraniol against AD, giving a reference to future experimental studies.

## Supplementary Information

**Additional file 1.** S1_Rare data for target identification, S2_Rare data for GO terms and KEGG pathways analyses, S3_Rare data for GO terms and KEGG pathway analyses of functional clustering, S4_Rare data for molecular docking.

## Data Availability

All data, models, and code generated or used during the study appear in the submitted article. No copyright is owned by individuals/organizations other than the authors.

## References

[1] Sonkusare SK, Kaul CL, Ramarao P (2005). Dementia of Alzheimer’s disease and other neurodegenerative disorders–memantine, a new hope. Pharmacol Res.

[2] Ballard C, Gauthier S, Corbett A, Brayne C, Aarsland D, Jones E (2011). Alzheimer’s disease. Lancet.

[3] Hardy JA, Higgins GA (1992). Alzheimer’s disease: the amyloid cascade hypothesis. Science.

[4] Sochocka M, Zwolińska K, Leszek J (2017). The infectious etiology of Alzheimer’s disease. Curr Neuropharmacol.

[5] Hu X, Wang T, Jin F (2016). Alzheimer’s disease and gut microbiota. Sci China Life Sci.

[6] Hampel H, Mesulam MM, Cuello AC, Farlow MR, Giacobini E, Grossberg GT, Khachaturian AS, Vergallo A, Cavedo E, Snyder PJ, Khachaturian ZS (2018). The cholinergic system in the pathophysiology and treatment of Alzheimer’s disease. Brain.

[7] Arshavsky YI (2020). Alzheimer’s disease: from amyloid to autoimmune hypothesis. Neuroscientist.

[8] Melnikova I (2007). Therapies for Alzheimer’s disease. Nat Rev Drug Discov.

[9] Hopkins AL (2008). Network pharmacology: the next paradigm in drug discovery. Nat Chem Biol.

[10] Deng XY, Xue JS, Li HY, Ma ZQ, Fu Q, Qu R, Ma SP (2015). Geraniol produces antidepressant-like effects in a chronic unpredictable mild stress mice model. Physiol Behav.

[11] Soliman SM, Sheta NM, Ibrahim BMM, El-Shawwa MM, Abd El-Halim SM (2020). novel intranasal drug delivery: geraniol charged polymeric mixed micelles for targeting cerebral insult as a result of ischaemia/reperfusion. Pharmaceutics.

[12] Atef MM, Emam MN, AboElGheit RE, Elbeltagi EM, Alshenawy HA, Radwan DA, Younis RL, Abd-Ellatif RN (2022). Mechanistic insights into ameliorating effect of geraniol on d-galactose induced memory impairment in rats. Neurochem Res.

[13] Baracchi D, Cabirol A, Devaud JM, Haase A, d'Ettorre P, Giurfa M (2020). Pheromone components affect motivation and induce persistent modulation of associative learning and memory in honeybees. Commun Biol.

[14] Guex N, Peitsch MC, Schwede T (2009). Automated comparative protein structure modeling with SWISS-MODEL and Swiss-PdbViewer: a historical perspective. Electrophoresis.

[15] Waterhouse A, Bertoni M, Bienert S, Studer G, Tauriello G, Gumienny R, Heer FT, de Beer TAP, Rempfer C, Bordoli L, Lepore R, Schwede T (2018). SWISS-MODEL: homology modelling of protein structures and complexes. Nucleic Acids Res.

[16] Rebhan M, Chalifa-Caspi V, Prilusky J, Lancet D (1998). GeneCards: a novel functional genomics compendium with automated data mining and query reformulation support. Bioinformatics.

[17] Bardou P, Mariette J, Escudié F, Djemiel C, Klopp C (2014). jvenn: an interactive Venn diagram viewer. BMC Bioinform.

[18] Thomas PD, Campbell MJ, Kejariwal A, Mi H, Karlak B, Daverman R, Diemer K, Muruganujan A, Narechania A (2003). PANTHER: a library of protein families and subfamilies indexed by function. Genome Res.

[19] Liao Y, Wang J, Jaehnig EJ, Shi Z, Zhang B (2019). WebGestalt 2019: gene set analysis toolkit with revamped UIs and APIs. Nucleic Acids Res.

[20] Shannon P, Markiel A, Ozier O, Baliga NS, Wang JT, Ramage D, Amin N, Schwikowski B, Ideker T (2003). Cytoscape: a software environment for integrated models of biomolecular interaction networks. Genome Res.

[21] Warde-Farley D, Donaldson SL, Comes O, Zuberi K, Badrawi R, Chao P, Franz M, Grouios C, Kazi F, Lopes CT, Maitland A, Mostafavi S, Montojo J, Shao Q, Wright G, Bader GD, Morris Q (2010). The GeneMANIA prediction server: biological network integration for gene prioritization and predicting gene functions. Nucleic Acids Res.

[22] Bader GD, Hogue CW (2003). An automated method for finding molecular complexes in large protein interaction networks. BMC Bioinform.

[23] Chin CH, Chen SH, Wu HH, Ho CW, Ko MT, Lin CY (2014). cytoHubba: identifying hub objects and sub-networks from complex interactome. BMC Syst Biol.

[24] Liu Y, Grimm M, Dai WT, Hou MC, Xiao ZX, Cao Y (2020). CB-Dock: a web server for cavity detection-guided protein-ligand blind docking. Acta Pharmacol Sin.

[25] Trott O, Olson AJ (2010). AutoDock Vina: improving the speed and accuracy of docking with a new scoring function, efficient optimization, and multithreading. J Comput Chem.

[26] Beard H, Cholleti A, Pearlman D, Sherman W, Loving KA (2013). Applying physics-based scoring to calculate free energies of binding for single amino acid mutations in protein-protein complexes. PLoS ONE.

[27] Vakalopoulos C (2017). Alzheimer’s disease: the alternative serotonergic hypothesis of cognitive decline. J Alzheimers Dis.

[28] Alfonso SI, Callender JA, Hooli B, Antal CE, Mullin K, Sherman MA, Lesné SE, Leitges M, Newton AC, Tanzi RE, Malinow R (2016). Gain-of-function mutations in protein kinase Cα (PKCα) may promote synaptic defects in Alzheimer’s disease. Sci Signal.

[29] Favit A, Grimaldi M, Nelson TJ, Alkon DL (1998). Alzheimer’s-specific effects of soluble beta-amyloid on protein kinase C-alpha and -gamma degradation in human fibroblasts. Proc Natl Acad Sci USA.

[30] Kinouchi T, Sorimachi H, Maruyama K, Mizuno K, Ohno S, Ishiura S, Suzuki K (1995). Conventional protein kinase C (PKC)-alpha and novel PKC epsilon, but not -delta, increase the secretion of an N-terminal fragment of Alzheimer's disease amyloid precursor protein from PKC cDNA transfected 3Y1 fibroblasts. FEBS Lett.

[31] Nicolas CS, Amici M, Bortolotto ZA, Doherty A, Csaba Z, Fafouri A, Dournaud P, Gressens P, Collingridge GL, Peineau S (2013). The role of JAK-STAT signaling within the CNS. JAKSTAT.

[32] Kanthasamy AG, Kitazawa M, Kanthasamy A, Anantharam V (2003). Role of proteolytic activation of protein kinase Cdelta in oxidative stress-induced apoptosis. Antioxid Redox Signal.

[33] Thal DM, Sun B, Feng D, Nawaratne V, Leach K, Felder CC, Bures MG, Evans DA, Weis WI, Bachhawat P, Kobilka TS, Sexton PM, Kobilka BK, Christopoulos A (2016). Crystal structures of the M1 and M4 muscarinic acetylcholine receptors. Nature.

