# Supplementary material for: Mechanistic insight of the potential of geraniol against Alzheimer’s disease
Source: Eur J Med Res. 2022 Jun 14;27:93. doi: 10.1186/s40001-022-00699-8 (PMC9199166; doi:10.1186/s40001-022-00699-8)
Supplement: Supplementary file 1 — Additional file 1. S1_Rare data for target identification, S2_Rare data for GO terms and KEGG pathways analyses, S3_Rare data for GO terms and KEGG pathway analyses of functional clustering, S4_Rare data for molecular docking. [file 40001_2022_699_MOESM1_ESM.zip › Supplementary materials/S4_Rare data for molecular docking /Affinity energy summary.pdf]

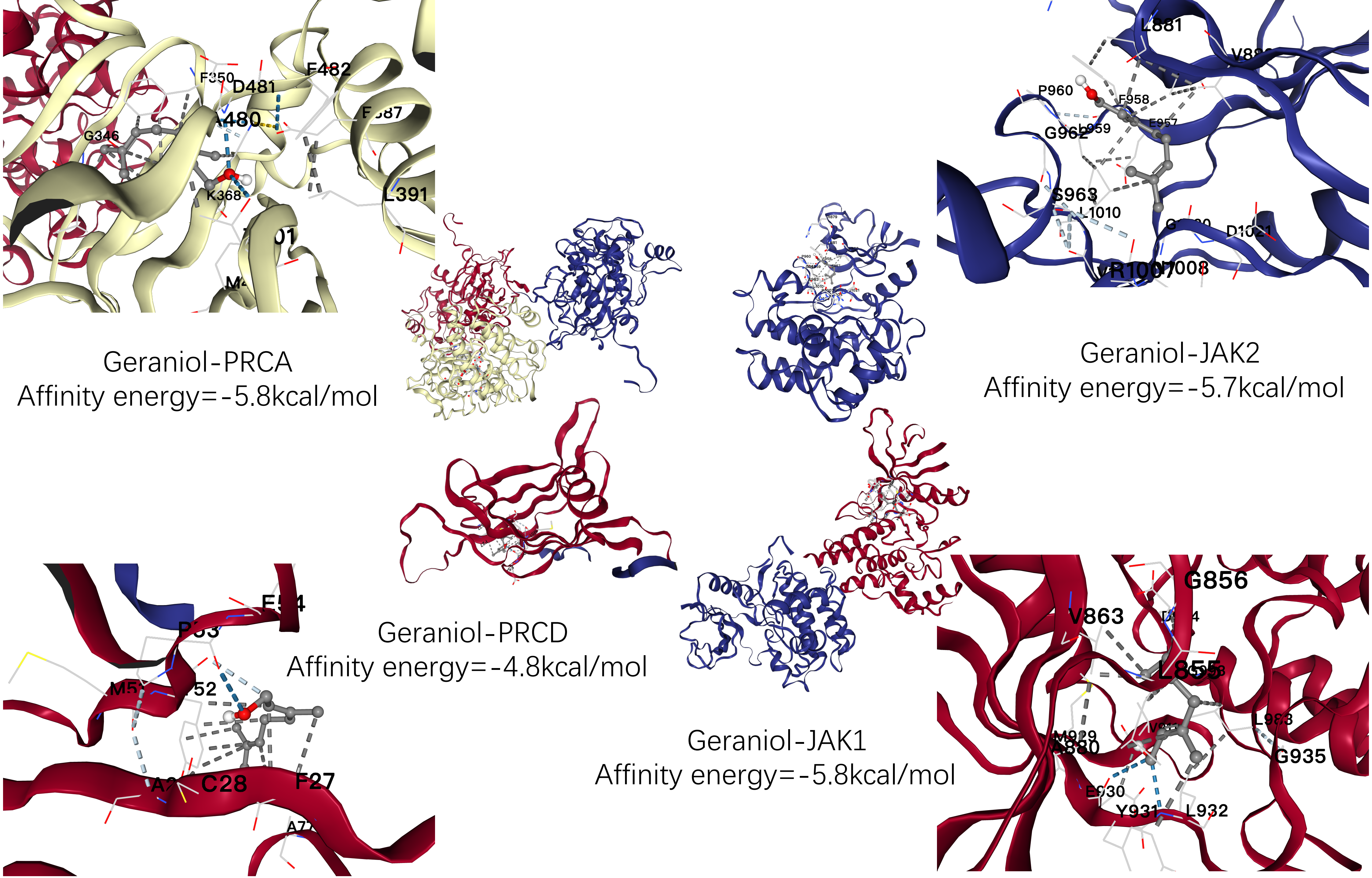
Geraniol-PRCA
Affinity energy=-5.8kcal/mol
Geraniol-JAK2
Affinity energy=-5.7kcal/mol
Geraniol-PRCD
Affinity energy=-4.8kcal/mol
Geraniol-JAK1
Affinity energy=-5.8kcal/mol
F482
D481
G346
K368
L391
L881
P960
F958
S963
D1021
C28
F27
G856
V863
G935
L932
