# Supplementary figures and images for: Mechanistic insight of the potential of geraniol against Alzheimer’s disease
Source: Eur J Med Res. 2022 Jun 14;27:93. doi: 10.1186/s40001-022-00699-8 (PMC9199166; doi:10.1186/s40001-022-00699-8)

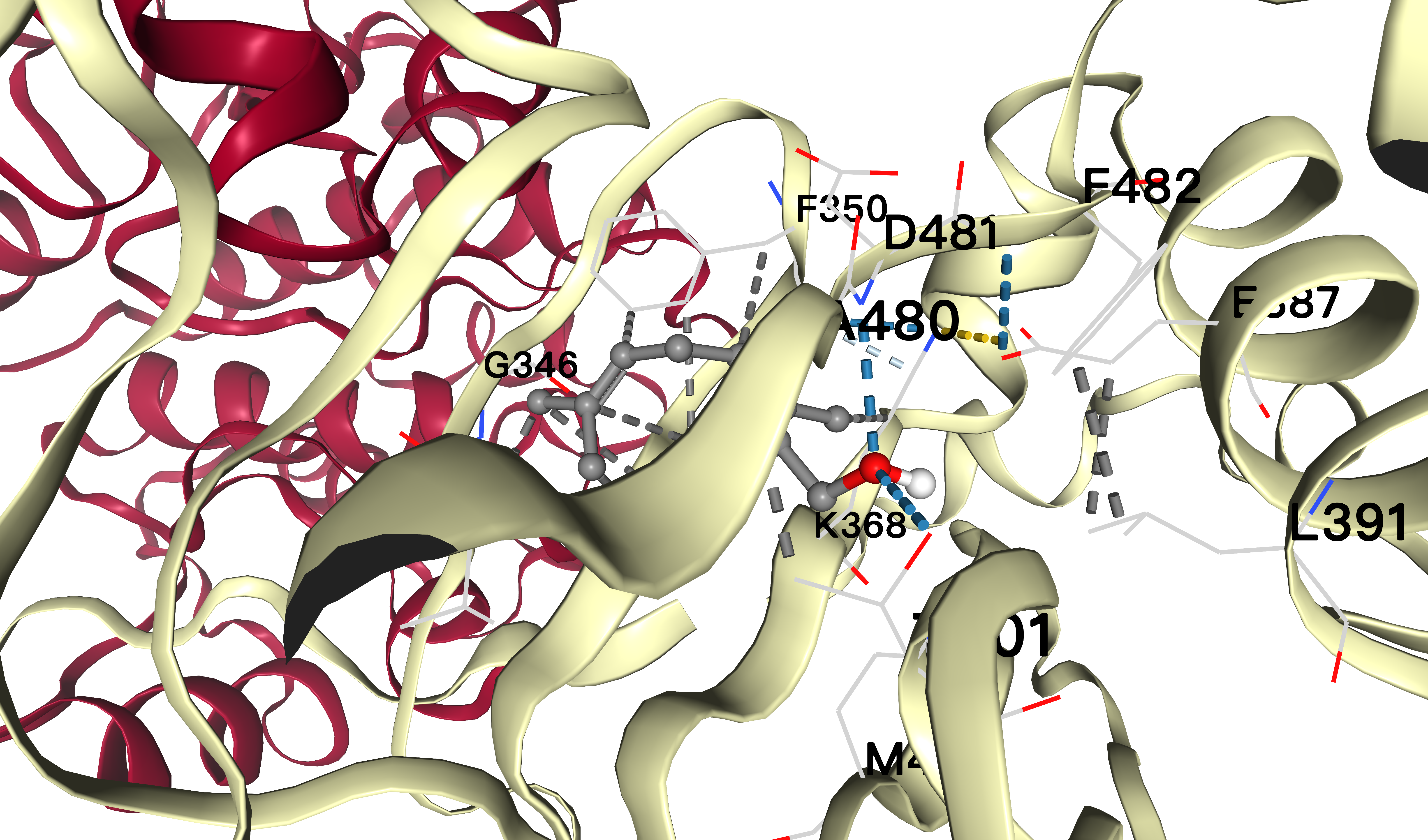

Supplement: Supplementary file 1 — Additional file 1. S1_Rare data for target identification, S2_Rare data for GO terms and KEGG pathways analyses, S3_Rare data for GO terms and KEGG pathway analyses of functional clustering, S4_Rare data for molecular docking. [file 40001_2022_699_MOESM1_ESM.zip › Supplementary materials/S4_Rare data for molecular docking /PRKCA-Geraniol complex /prkca (1).png]

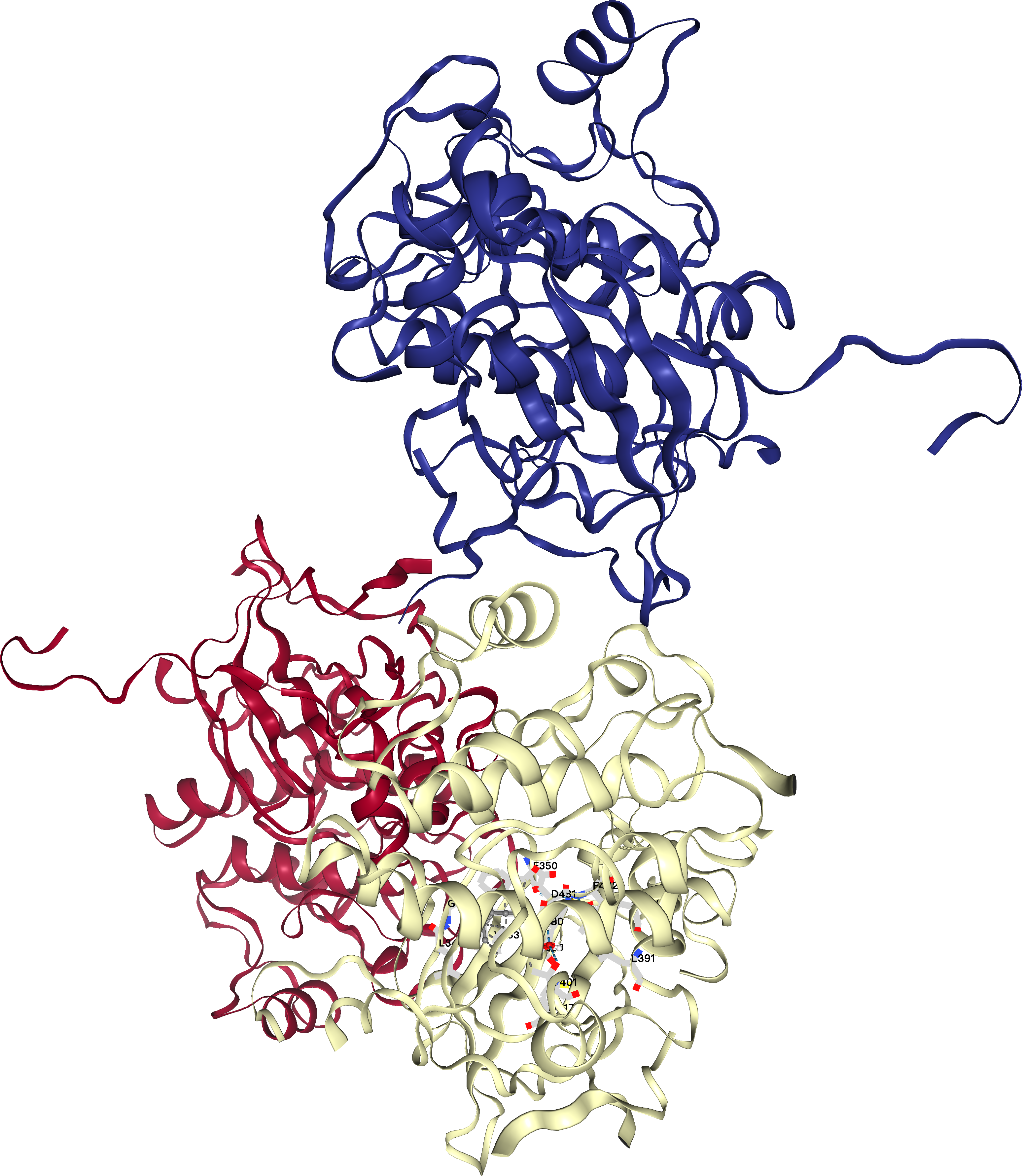

Supplement: Supplementary file 1 — Additional file 1. S1_Rare data for target identification, S2_Rare data for GO terms and KEGG pathways analyses, S3_Rare data for GO terms and KEGG pathway analyses of functional clustering, S4_Rare data for molecular docking. [file 40001_2022_699_MOESM1_ESM.zip › Supplementary materials/S4_Rare data for molecular docking /PRKCA-Geraniol complex /prkca.png]

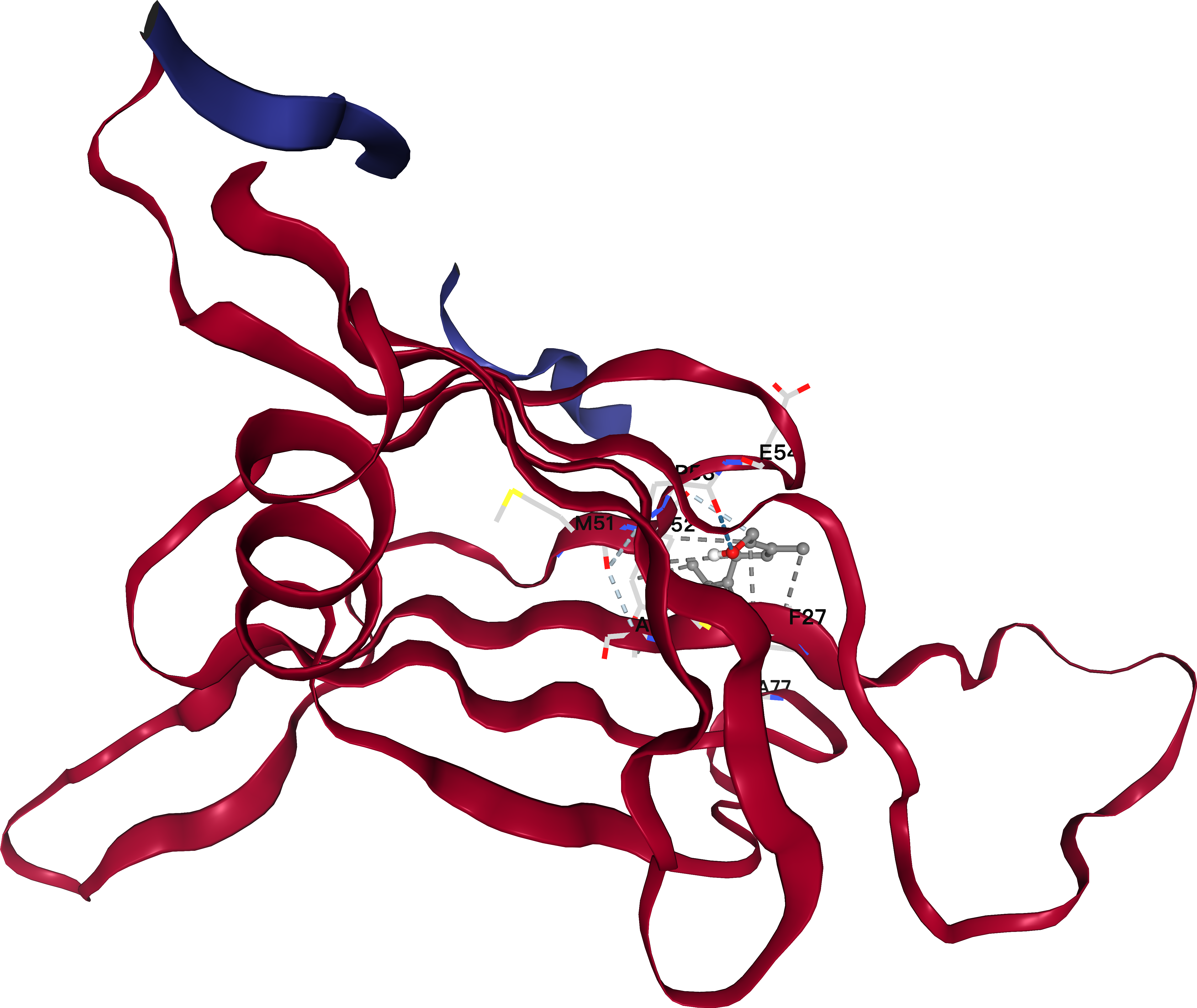

Supplement: Supplementary file 1 — Additional file 1. S1_Rare data for target identification, S2_Rare data for GO terms and KEGG pathways analyses, S3_Rare data for GO terms and KEGG pathway analyses of functional clustering, S4_Rare data for molecular docking. [file 40001_2022_699_MOESM1_ESM.zip › Supplementary materials/S4_Rare data for molecular docking /PRKCD-Geraniol complex /prkcd.png]

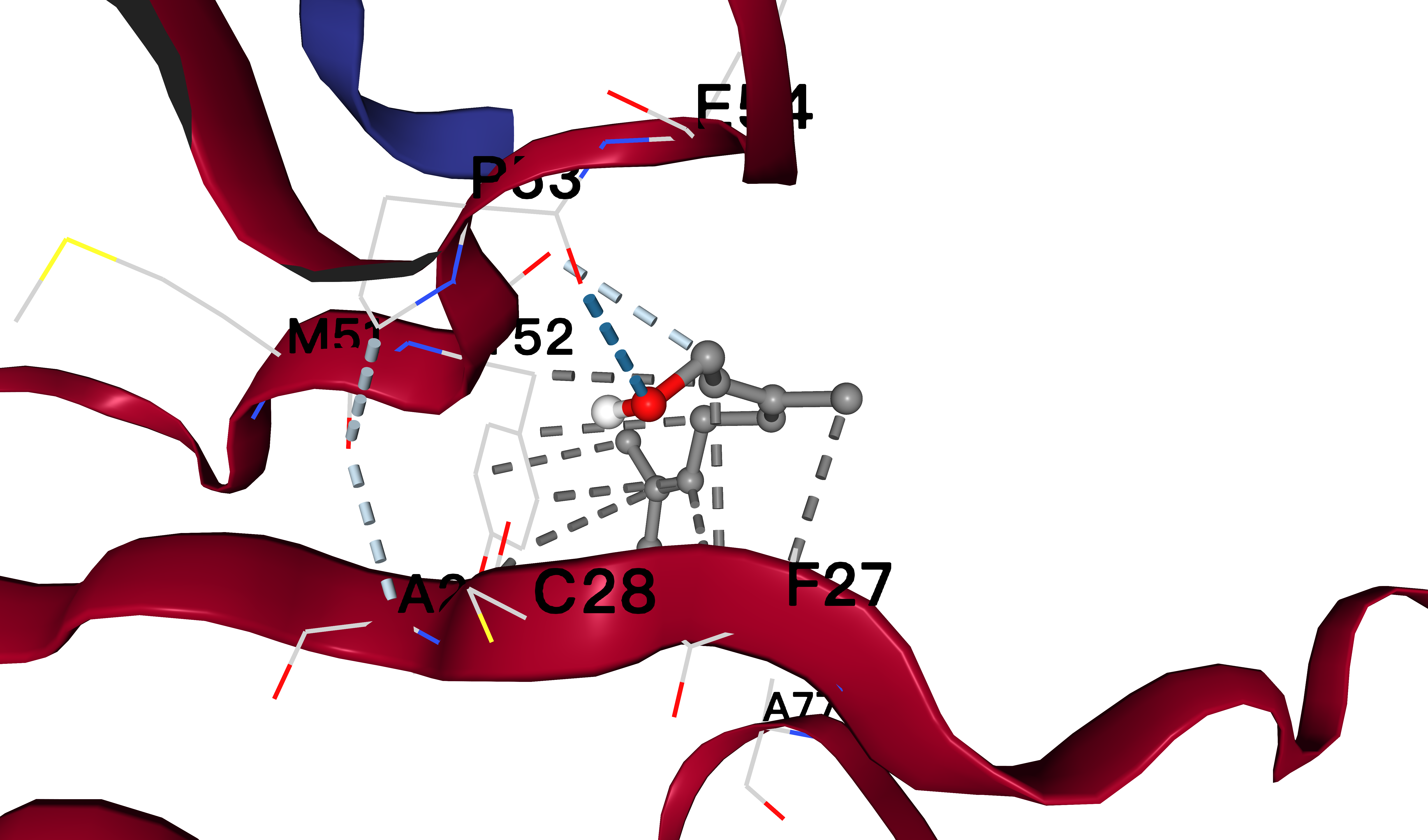

Supplement: Supplementary file 1 — Additional file 1. S1_Rare data for target identification, S2_Rare data for GO terms and KEGG pathways analyses, S3_Rare data for GO terms and KEGG pathway analyses of functional clustering, S4_Rare data for molecular docking. [file 40001_2022_699_MOESM1_ESM.zip › Supplementary materials/S4_Rare data for molecular docking /PRKCD-Geraniol complex /prkcd (1).png]

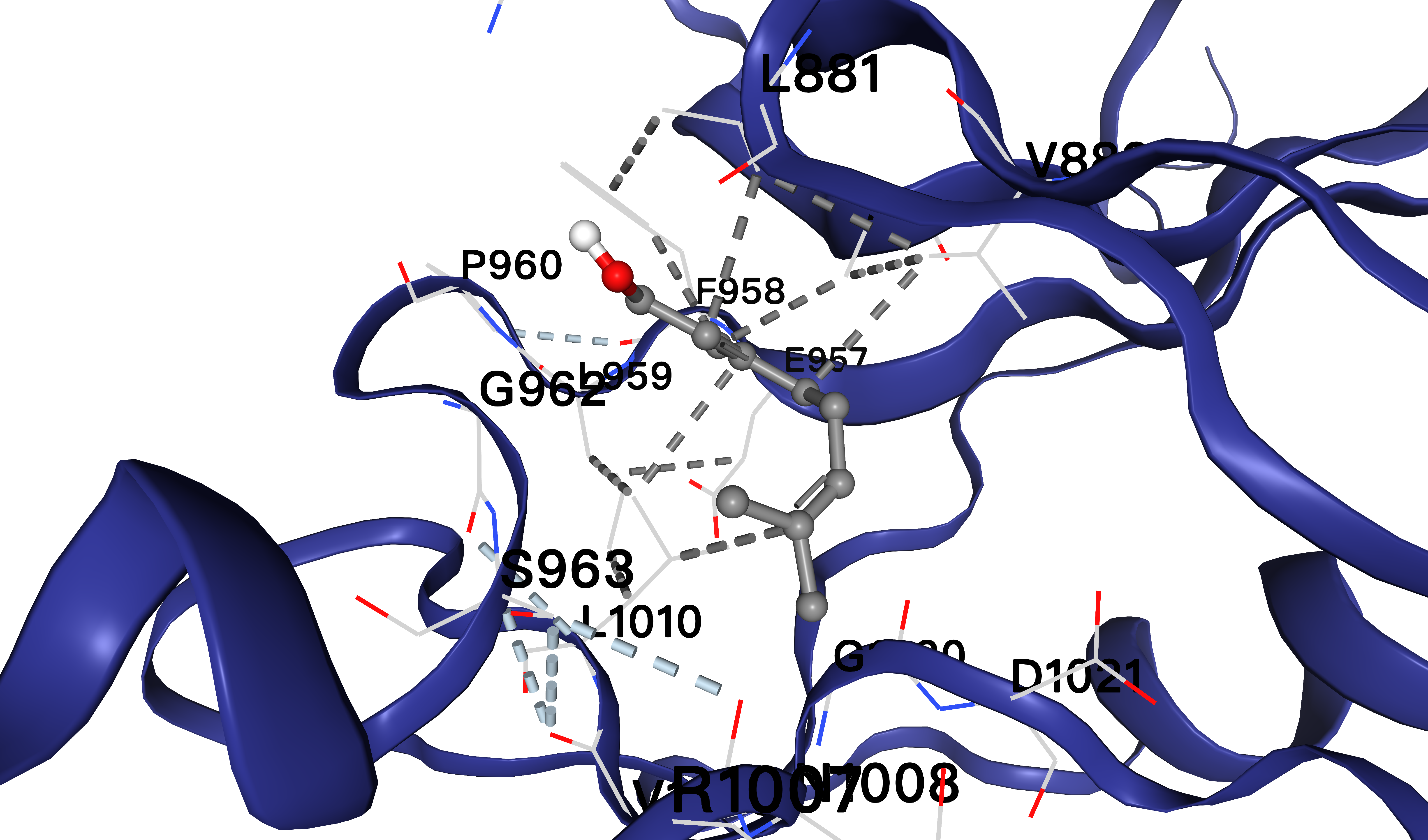

Supplement: Supplementary file 1 — Additional file 1. S1_Rare data for target identification, S2_Rare data for GO terms and KEGG pathways analyses, S3_Rare data for GO terms and KEGG pathway analyses of functional clustering, S4_Rare data for molecular docking. [file 40001_2022_699_MOESM1_ESM.zip › Supplementary materials/S4_Rare data for molecular docking /JAK1-Geraniol complex /jak1 (1).png]

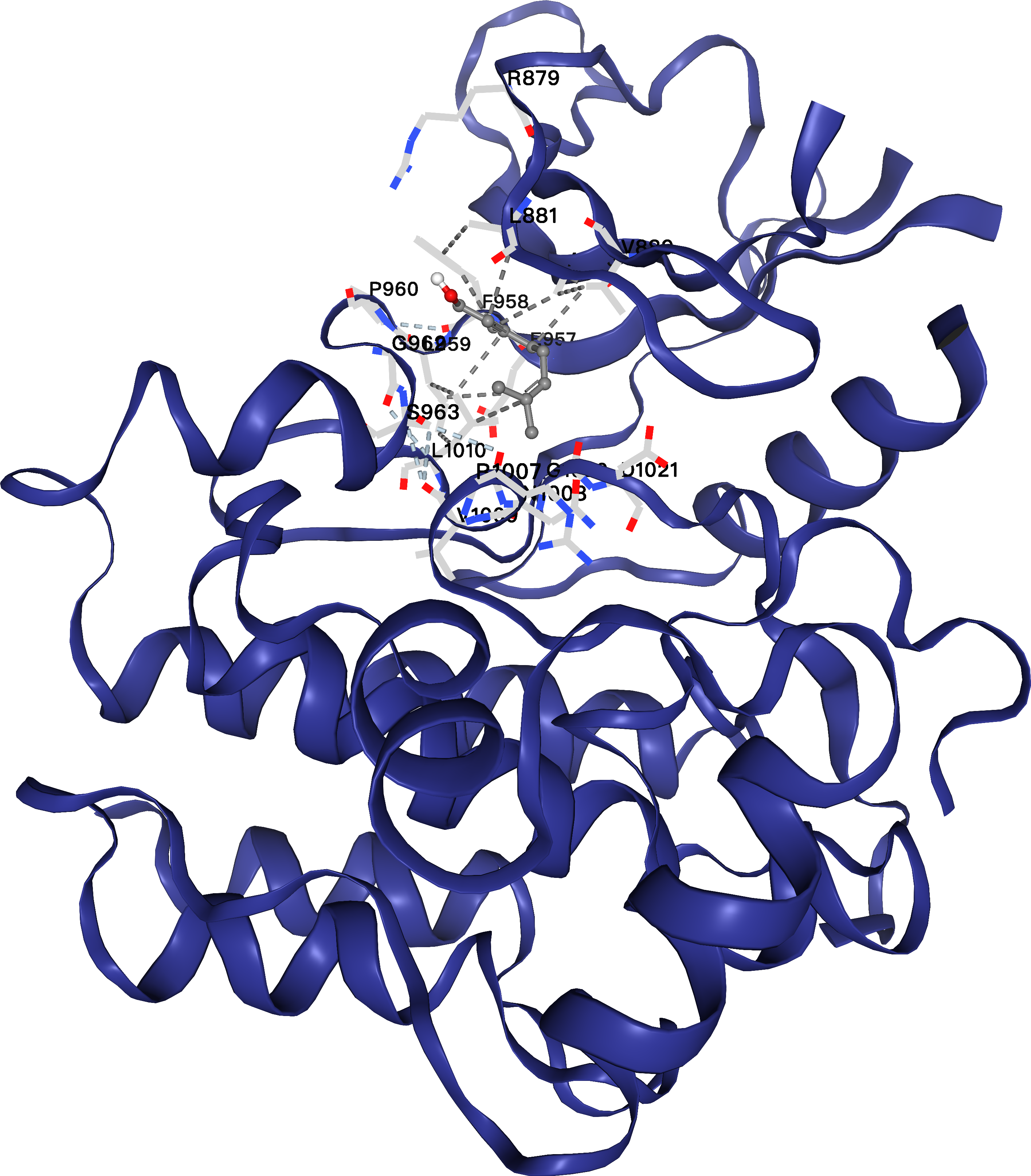

Supplement: Supplementary file 1 — Additional file 1. S1_Rare data for target identification, S2_Rare data for GO terms and KEGG pathways analyses, S3_Rare data for GO terms and KEGG pathway analyses of functional clustering, S4_Rare data for molecular docking. [file 40001_2022_699_MOESM1_ESM.zip › Supplementary materials/S4_Rare data for molecular docking /JAK1-Geraniol complex /jak1.png]

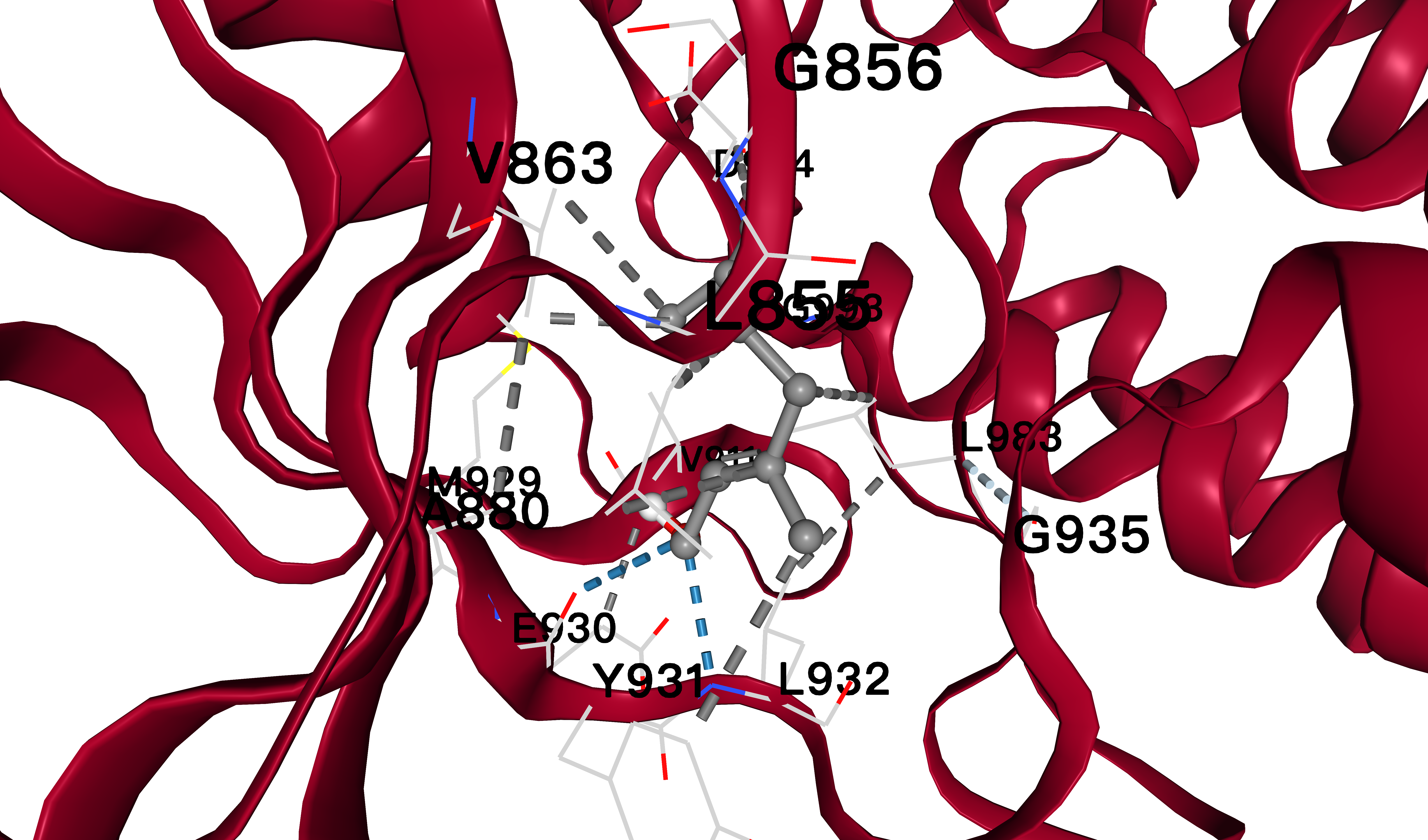

Supplement: Supplementary file 1 — Additional file 1. S1_Rare data for target identification, S2_Rare data for GO terms and KEGG pathways analyses, S3_Rare data for GO terms and KEGG pathway analyses of functional clustering, S4_Rare data for molecular docking. [file 40001_2022_699_MOESM1_ESM.zip › Supplementary materials/S4_Rare data for molecular docking /JAK2-Geraniol complex /jak2 (1).png]

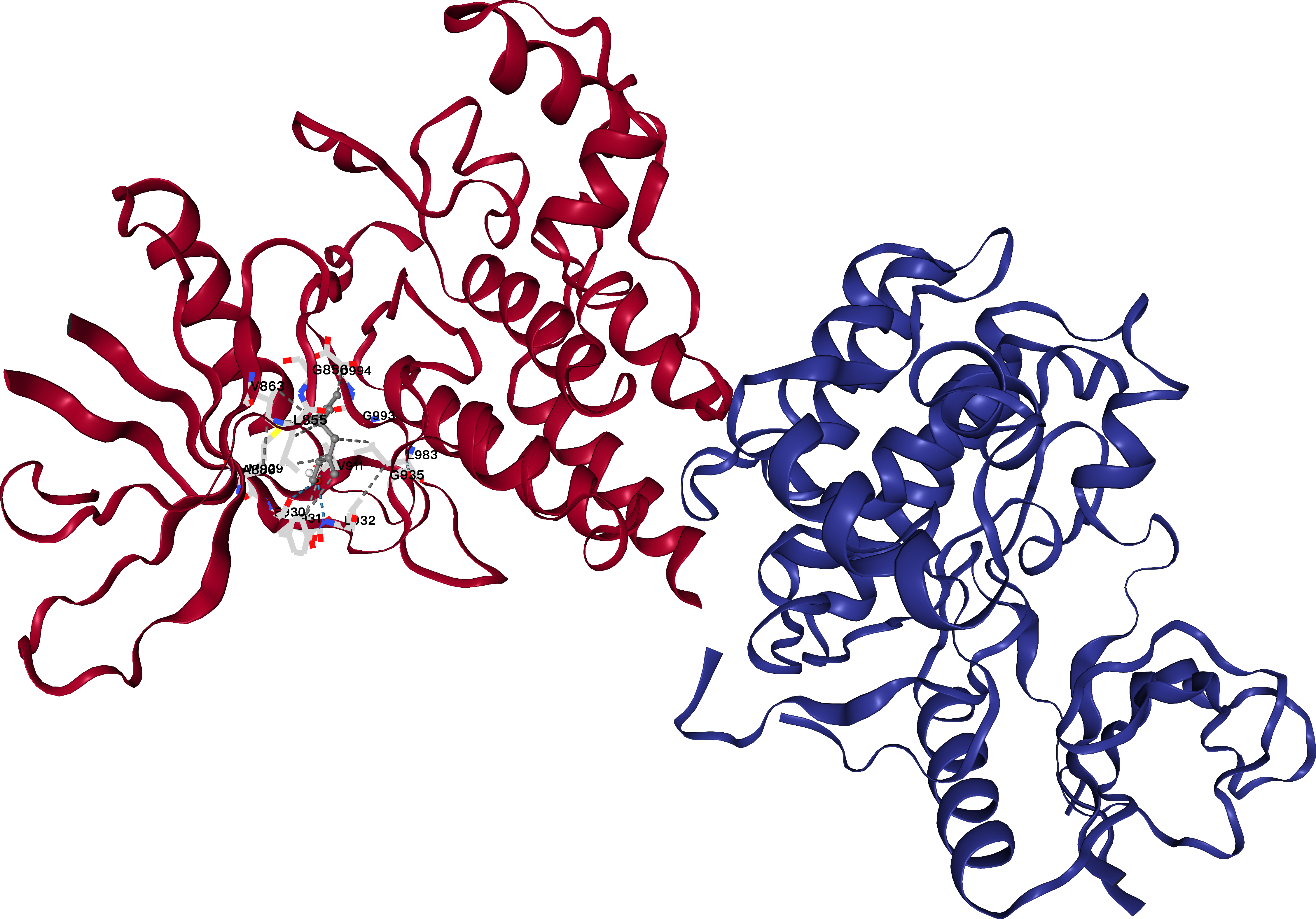

Supplement: Supplementary file 1 — Additional file 1. S1_Rare data for target identification, S2_Rare data for GO terms and KEGG pathways analyses, S3_Rare data for GO terms and KEGG pathway analyses of functional clustering, S4_Rare data for molecular docking. [file 40001_2022_699_MOESM1_ESM.zip › Supplementary materials/S4_Rare data for molecular docking /JAK2-Geraniol complex /jak2.png]
